# Long-term outcome of severe herpes simplex encephalitis: a population-based observational study

**DOI:** 10.1186/s13054-015-1046-y

**Published:** 2015-09-21

**Authors:** Youenn Jouan, Leslie Grammatico-Guillon, Fabien Espitalier, Xavier Cazals, Patrick François, Antoine Guillon

**Affiliations:** Service de Réanimation Polyvalente, CHRU Tours, 2 boulevard Tonnellé, 37000 Tours, France; Service d’information médicale, d’épidémiologie et d’économie de la santé, UREH, EE EES, Hôpital Bretonneau, CHRU Tours, 2 boulevard Tonnellé, 37000 Tours, France; Faculté de médecine, Université François Rabelais, 10 boulevard Tonnellé, 37032 Tours, France; Département d’Anesthésie & Réanimation, Hôpital Trousseau, CHRU Tours, 2 boulevard Tonnellé, 37000 Tours, France; Service de Neuroradiologie, Hôpital Bretonneau, CHRU Tours, 2 boulevard Tonnellé, 37000 Tours, France; Service de Neurochirurgie, Hôpital Bretonneau, CHRU Tours, 2 boulevard Tonnellé, 37000 Tours, France

## Abstract

**Introduction:**

Herpes simplex encephalitis (HSE) is a rare disease with a poor prognosis. No recent evaluation of hospital incidence, acute mortality and morbidity is available. In particular, decompressive craniectomy has rarely been proposed in cases of life-threatening HSE with temporal herniation, in the absence of evidence. This study aimed to assess the hospital incidence and mortality of HSE, and to evaluate the characteristics, management, the potential value of decompressive craniectomy and the outcome of patients with HSE admitted to intensive care units (ICUs).

**Methods:**

*Epidemiological study*: we used the hospital medical and administrative discharge database to identify hospital stays, deaths and ICU admissions relating to HSE in 39 hospitals, from 2010 to 2013. *Retrospective monocentric cohort*: all patients with HSE admitted to the ICU of the university hospital during the study were included. The use of decompressive craniectomy and long-term outcome were analyzed. The initial brain images were analyzed blind to outcome.

**Results:**

The hospital incidence of HSE was 1.2/100,000 inhabitants per year, 32 % of the patients were admitted to ICUs and 17 % were mechanically ventilated. Hospital mortality was 5.5 % overall, but was as high as 11.9 % in ICUs. In the monocentric cohort, 87 % of the patients were still alive after one year but half of them had moderate to severe disability. Three patients had a high intracranial pressure (ICP) with brain herniation and eventually underwent decompressive hemicraniectomy. The one-year outcome of these patients did not seem to be different from that of the other patients. It was not possible to predict brain herniation reliably from the initial brain images.

**Conclusions:**

HSE appears to be more frequent than historically reported. The high incidence we observed probably reflects improvements in diagnostic performance (routine use of PCR). Mortality during the acute phase and long-term disability appear to be stable. High ICP and brain herniation are rare, but must be monitored carefully, as initial brain imaging is not useful for identifying high-risk patients. Decompressive craniectomy may be a useful salvage procedure in cases of intractable high ICP.

**Electronic supplementary material:**

The online version of this article (doi:10.1186/s13054-015-1046-y) contains supplementary material, which is available to authorized users.

## Introduction

Herpes simplex encephalitis (HSE) is a rare but severe condition. In the absence of treatment, prognosis is extremely poor, with a mortality rate of about 70 % [1]. Improvements in diagnostic techniques (based on use of the polymerase chain reaction, PCR) and the advent of acyclovir treatment decreased mortality rate to 15–20 % [2, 3]. However, recent epidemiological data are lacking. For example, incidence was evaluated at about 1 case per 1,000,000 inhabitants per year during the 1990s, but PCR was not routinely used at the time [2]. Moreover, although deaths occurring in the long term due to complications have been well described [2, 3], deaths occurring in the acute phase have been little analyzed. The studies published to date do not focus on the most severe cases. Few data are available for the most severe cases of HSE, those admitted to intensive care units (ICUs), particularly as concerns their epidemiology, initial management, acute-phase mortality, and long-term outcome. The notion that high intracranial pressure (ICP) might be associated with a poor acute-phase outcome of severe cases of encephalitis arose from the historical series of Barnett et al. [4]. High ICP is a well-known complication of acute bacterial meningitis [5, 6] and has also been reported in cases of viral meningitis [7]. Uncontrolled high ICP may lead to a decrease in cerebral blood flow, potentially resulting in fatal brain herniation. Medical strategies should be considered for the management of patients with high ICP due to HSE, and such approaches are now well established, based on many years of experience with patients with traumatic brain injury [8]. However, drug-based strategies targeting ICP may be ineffective, and salvation decompressive hemicraniectomy has been proposed and cases reported [9–18]. There are currently no recommendations supporting this strategy, and obtaining evidence to support its use would be difficult, given the rarity of this clinical condition and potential ethical problems. Nevertheless, care providers should be aware of this possible lifesaving procedure and the circumstances in which it might be appropriate. More detailed data are thus required, with better characterization and description of the management of the most severe cases of HSE.

In this study, we aimed to assess the epidemiological and clinical features of severe HSE, and to describe its management and long-term neurological outcome.

## Methods

### Case definition

Diagnosis of HSE was validated with the association of: 1) diagnostic criteria for encephalitis and 2) a positive PCR finding for herpes simplex in cerebrospinal fluid (CSF). Encephalitis was therefore defined [19] with altered mental status (defined as decreased or altered level of consciousness, lethargy or personality change) lasting more than 24 h with no alternative cause identified, and at least three of the following criteria: documented fever ≥38 °C within the 72 h before or after presentation, generalized or partial seizures not fully attributable to a preexisting seizure disorder, new onset of focal neurologic findings, CSF white blood count ≥5/mm^3^, abnormality of brain parenchyma on neuroimaging suggestive of encephalitis that is either new from prior studies or appears acute in onset, abnormality on electroencephalography that is consistent with encephalitis and not attributable to another cause.

### Epidemiological study

#### Study location and duration

A retrospective study was performed from 2010 to 2013, in a large, representative region of France (2.5 million inhabitants, including rural and urban areas), with one university hospital (2,000 beds and approximately 90,000 admissions in the emergency department annually), one regional hospital and 37 general and private hospitals. HSE incidence rates were calculated using the number of stays associated with in-hospital HSE as the numerator, and annual population estimates as the denominator [20].

#### Data retrieval and patient selection

We retrospectively screened the French national hospital discharge database *Programme de Médicalisation des Systèmes d’Information* (PMSI). The PMSI database provides medical and administrative data for each hospital stay in France, available since 1997. These data are collected for each French hospital stay, in public and private sectors of hospitalization. Data are anonymized and linked, such that stays are linked to patients due to a unique anonymous number, providing follow-up for consecutive hospital stays for each patient. PMSI is becoming a useful and powerful epidemiologic tool [21–23]. We used the regional PMSI database to identify all hospital stays related to HSE from 2010 to 2013, extracting hospital stays with the specific International Classification of Disease, Tenth Revision (ICD-10) diagnostic codes for *viral meningitis* (A87), *viral encephalitis* (A85, A86) and *herpetic encephalitis* (B00.3). We linked the data for multiple hospitalizations to those for the patients concerned, using the encrypted patient number, to obtain the patient database. We studied a 4-year period, so as to obtain a more accurate estimation of HSE incidence. Patients under the age of 18 years were not included in this study.

#### Validation of the case definition

HSE case definition was validated in two steps, using a sample of hospital charts from the university hospital as the gold standard. We first assessed the reliability of case definition by verifying medical files of all patients with HSE admitted to the university hospital during the study period selected with the coding algorithm (n=26). All the records reviewed related to true cases of HSE, giving a positive predictive value (PPV) of 100 %. In the second stage of validation, we determined the reliability of case definition for 200 patients without codes relating to HSE, screened for the presence of an infectious syndrome and neurological involvement in the form of coma/convulsions and admitted to the university hospital. All the records obtained corresponded to true negatives, giving a negative predictive value (NPV) of the case definition of 100 %.

### Analysis of a monocentric series of severe HSE cases

The characteristics, initial management and long-term neurological outcome of patients with severe HSE hospitalized at the university hospital during the study period were reviewed retrospectively. Severe HSE was defined on the basis of the PMSI algorithm associated with admission in ICU. We analyzed patient characteristics, initial management and long-term neurological outcome over the 4-year study period. We also described the situations in which the decision was taken to perform a decompressive hemicraniectomy.

#### Data retrieval

We analyzed the medical files of the selected patients, focusing on the following: the reason for ICU admission, CSF findings (including the results of PCR for herpes simplex virus, (HSV)), and the occurrence of coma (defined by Glasgow Coma Scale (GCS) score <8) requiring mechanical ventilation. The time from the onset of symptoms to the initiation of acyclovir treatment was also noted. A Sequential Organ Failure Assessment (SOFA) score was calculated 24 h after admission to the ICU. The technique used for brain imaging (computed tomography (CT) or magnetic resonance imaging (MRI)) and the time between the onset of symptoms and brain imaging were reported. We evaluated the occurrence of high ICP by examining medical records, and that of brain herniation through a review of all brain images. Two neuroradiologists blind to the identity of the patients and their outcomes retrospectively reanalyzed the brain images. Volumetric analyses of the lesions were carried out with iPlan® Net v.3.5.0 (Brainlab, Munich, Germany). The neuroradiologists were independently asked to determine whether there was a significant risk of brain herniation from the first brain image obtained for each patient (they were asked to reply “yes” or “no”). We then determined the sensitivity, specificity, positive and negative predictive values of the first brain image for predicting brain herniation. The therapeutic strategies used to manage brain herniation were recorded: medical treatments (heavy sedation with barbiturates, use of osmotherapy) and the suggestion and performance of salvage decompressive hemicraniectomy.

#### Patient outcome

Long-term neurological outcome was assessed with the Glasgow Outcome Scale (GOS) 1 year after hospital discharge. If post acute care follow-up was provided only out from the hospital, the GOS was assessed by interviewing the patient’s general practitioner (GP).

#### Statistical analysis

Numerical results are expressed as medians and interquartile ranges. Statistical tests were not carried out for group comparisons, due to the small sample size. Instead, we provide readers with all the individual data. Spearman’s rank correlation test was used to assess the correlation between variables. Statistical analysis was performed with GraphPad Prism® v.6.0 (GraphPad Software, San Diego, CA, USA), and *p* values <0.05 were considered statistically significant.

### Ethical approval

The treatment of personal health data of this observational research has been approved by the *Commission Nationale de l’Informatique et des Libertés* (CNIL), in compliance with the Helsinki Declaration. No consent was needed from the patients involved in this retrospective study.

## Results

### Epidemiological study

During the study period, 1561 patients were hospitalized for viral meningitis or encephalitis and 128 patients were hospitalized for HSE. The annual hospitalization rate for HSE was 1.2 per 100,000 inhabitants per year. For viral meningitis, overall hospital incidence was 16.1 per 100,000 inhabitants per year.

**Fig. 1:**
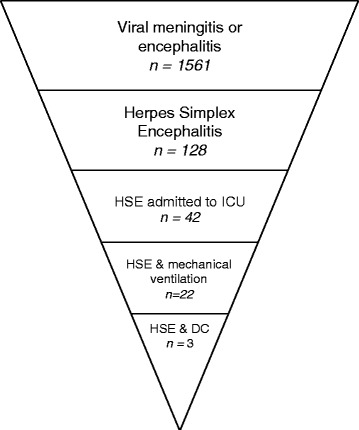
Diagram showing the retrospective screening process from viral encephalitis to severe HSE with brain herniation. A retrospective screening of the PMSI medical database was carried out to identify hospital stays related to HSE, from 2010 to 2013, in a large region of France (2.5 million inhabitants and 39 hospital centers) on the basis of International Classification of Diseases, Tenth Revision (ICD-10)-specific related diagnosis codes (viral meningitis and encephalitis, herpes simplex encephalitis, admission to ICU, mechanical ventilation, decompressive craniectomy). *DC* decompressive craniectomy, *HSE* herpes simplex encephalitis, *ICU* intensive care unit, *PMSI,* *Programme de Médicalisation des Systèmes d’Information*

Over the study period, 32.8 % of the 128 HSE patients were admitted to an ICU during their hospital stay for HSE, and 52.4 % of these patients were mechanically ventilated (corresponding to 17 % of the total population of patients with HSE). Overall in-hospital mortality was 5.5 %, but mortality rates were as high as 11.9 % for ICUs (5/42) (Fig. 1).

### Analysis of the monocentric series of severe HSE

During the study period, 549 stays at the university hospital were coded as related to *viral meningitis* or *viral encephalitis* according to the ICD-10 codes. These hospitals stays corresponded to 498 individual patients (several of whom had multiple hospital stays). Twenty-six of these patients had HSE, and 14 were hospitalized in ICUs. Patients were admitted to ICUs for changes in mental state and/or meningitis symptoms, and the lowest GSC score during the first 48 h after admission was 9 [7; 13]. None of the patients had signs of shock at admission. Ten patients became comatose, either progressively (*n* = 6) or after seizures (*n* = 4), and were intubated and mechanically ventilated. Thus, 38.5 % of all patients admitted for HSE in the university hospital required invasive mechanical ventilation. On CSF analysis, the median leukocyte count was 83 [22; 380] per mm^3^, and the median protein concentration was 0.9 [0.6; 1.0] g/L. Three of the 14 patients had fewer than 10 leukocytes/mm^3^ on the initial CSF analysis. All PCRs performed on CSF were positive for HSV: HSV-1 in 13 cases and HSV-2 in the remaining case. The median time from symptom onset to treatment was 6 [4; 7] days. The median duration of mechanical ventilation was 13 [6; 19] days. Sedatives were used for a median of 4 [1; 8] days. Six patients required vasopressors over a median of 8 [6; 10] days. Median SOFA score 24 h after admission was 4 [1; 5].

#### Patient characteristics and outcome

Two of the 14 patients hospitalized in the ICU for HSE died during their stay in the ICU. No other deaths were noted after 1 year of follow-up. Thus, the 1-year specific mortality (GOS 1) was 14.3 %. The remaining GOS scores at 1 year were distributed as follow: 0 % GOS 2, 21 % GOS 3, 21 % GOS 4 and 43 % GOS 5. For all patients, follow-up after discharge was carried out by the university hospital, except for two patients who were initially managed by the university hospital and then by their GP, and two who were managed only by their GP. The detailed characteristics of the 14 patients initially hospitalized in ICU are provided in Additional file 1.

#### Comparison of patients with and without brain herniation

**Fig. 2:**
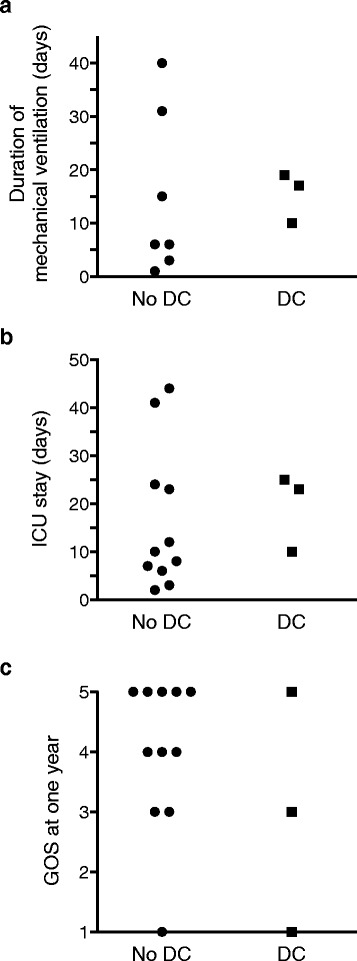
Comparison of outcomes according to craniectomy status (decompressive hemicraniectomy versus no decompressive hemicraniectomy) for (**a**) duration of mechanical ventilation (days); (**b**) length of stay in the ICU (days); and (**c**) Glasgow Outcome Scale (GOS) at one year. *DC* decompressive craniectomy, *ICU* intensive care unit

High ICP was suspected after neurological deterioration in three patients hospitalized in the ICU for HSE, and temporal herniation of the brain was diagnosed on CT scans. There was no clinical or radiological evidence of brain herniation or high ICP for the other 11 patients. The principal characteristics of the patients are presented in Table 1, according to presence or absence of brain herniation. SOFA scores 24 h after admissions were higher in patients with brain herniation than in those without brain herniation. The management of patients with brain herniation was complicated by refractory intracranial hypertension. Optimal medical treatments to decrease intracranial pressure were administered for 2 to 24 h but failed, and all patients eventually underwent decompressive hemicraniectomy as a salvage therapy. None of the patients received corticosteroids. The characteristics and management of the patients with decompressive hemicraniectomy are presented in Additional file 2. No direct complication of decompressive hemicraniectomy was noted. A comparison of the outcomes of patients with and without decompressive hemicraniectomy, in terms of the duration of mechanical ventilation, length of stay in the ICU and GOS at 1 year, is presented in Fig. 2. No significant difference in 1-year outcome was observed between the two groups (Fig. 2c). One of the patients that underwent salvage decompressive hemicraniectomy died in the ICU, one had cognitive impairment and frontal syndrome, and the third had normal cognitive function at 1 year.

**Table 1:** Comparison of patients hospitalized in ICUs for HSE with and without brain herniation

	Patients without brain herniation	Patients with brain herniation
Number of patients	11	3
Age (year)	48 [31;70]	58 [45;69]
Sex (male, %)	55	33
Time from symptom onset to first brain scan (days)	5 [3;6]	5 [4;6]
Time from symptom onset to the start of treatment (days)	6 [4;7]	5 [5;6]
SOFA 24 h after admission	2 [1;4]	6 [5;7]
Lowest GCS during the first 48 h after admission	10 [9;144]	7 [7;7]
Patients with seizures (%)	27.3	33.3
Mechanical ventilation (%)	64	100
Decompressive craniectomy (*n*)	0	3

*SOFA* Sequential Organ Failure Assessment, *GCS* Glasgow Coma Scale

#### Brain imaging

**Fig. 3:**
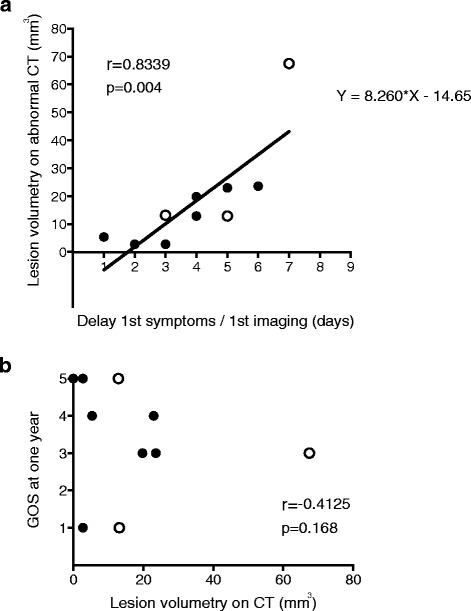
First brain imaging after admission. **a** Lesion volumetry (mm^3^) measured on CT scans, as a function of time from symptom onset to the procedure (in days). **b** Glasgow Outcome Scale (GOS) score at 1 year, as a function of lesion volumetry (mm^3^) results for CT scans. Patients with brain herniation and high intracranial pressure treated by decompressive craniectomy are represented as *white dots. CT* computed tomography

All patients underwent brain imaging, a median of 5 [3; 6] days after the onset of symptoms. Twelve of the 14 initial brain scans revealed lesions in the temporal, frontal or insular regions. The other two were normal (performed 5 and 9 days after the onset of symptoms). CT was the first type of brain imaging carried out in 12 patients, whereas the other two underwent MRI first. Given the interpatient variation in time to first imaging procedure and the rapid progression of HSV lesions in the absence of treatment, we present the brain volumetry results according to the time between symptom onset and the performance of CT (Fig. 3a): for abnormal CT scans, there was a positive correlation between lesion volumetry results and the time from the onset of symptoms to the performance of the first imaging procedure (*r* = 0.834; *p* = 0.004). No correlation was found between 1-year GOS and lesion volumetry results for the first CT scan (Fig. 3b). The prediction of brain herniation by neuroradiologists, based on the first scan, had a sensitivity of 50 %, a specificity of 82 %, a positive predictive value of 43 % and a negative predictive value of 86 %. Details concerning the differences between initial CT and MRI findings are provided in Additional file 3.

## Discussion

This study is the first, to our knowledge, to report the incidence and long-term follow-up results for patients with severe HSE since the introduction of routine PCR testing. The incidence of HSE due to confirmed HSV-1 and HSV-2 infections was 1.2 cases per 100,000 inhabitants per year, about ten times higher than that reported at the end of the 20th century [2]. Presumably, the observed difference reflects improvements in diagnostic performance (routine use of PCR) over time rather than an absolute increase in the rate of infection.

HSE accounted for 8.2 % of encephalitis hospitalization, which is slightly lower than the rate reported in a recent study [24] using administrative database from the United States, in which HSE accounted for 13.8 % of encephalitis hospitalization.

The high frequency of admission of HSE patients to ICU (32 %) and of the need for mechanical ventilation (17 %) highlight the need to get intensive care specialists more involved in the management of these patients. Furthermore, the overall in-hospital mortality was 5.5 % (at a level similar to the crude mortality observed in the United States: 8.9 % [24]), but increased up to 12 % for patients admitted in the ICU. This specific data has rarely been reported until now. For patients with all-cause encephalitis admitted to the ICU, mortality was 17 to 18 % in two recent studies [25, 26] and no clear differences were observed in short-term outcome between infectious and noninfectious (autoimmune) etiologies.

Regarding the 1-year follow up of the university hospital ICU cohort, all deaths occurred in the ICU, highlighting again the importance of initial management for the most severe cases. Hjalmarsson et al*.* [2] reported a similar 12 % 1-year mortality, but in a non-ICU-focused cohort. In the retrospective cohort studied by Raschilas et al*.* [3], in which 71 % of the patients were admitted to the ICU, 1-year mortality was about double that reported here (28 %).

The incidence of disability after HSE was high, with only 50 % of the patients displaying complete neurologic recovery at 1 year. This finding is similar to other observations [2, 3, 27] and highlights the burden of this infection, long after discharge.

We also investigated the use of decompressive hemicraniectomy to treat life-threatening HSE and the 1-year outcome for patients undergoing this intervention. The use of decompressive hemicraniectomy remains exceptional in HSE management, and there is currently no evidence of its efficacy. Only case reports have been published to date ([9–18], Additional file 4). The overall success rate for the published cases is surprisingly high: 10 of 13 cases in adults resulted in complete neurologic recovery. However, this undoubtedly reflects a nonpublication bias: no case report of severe HSE with high ICP and brain herniation treated medically has been published, and there have also been no reports of decompressive hemicraniectomy failure. We analyzed all patients admitted for severe HSE in the university hospital and we identified no case of high ICP successfully treated with medical therapies only. Furthermore, those undergoing decompressive hemicraniectomy were the patients with the most severe symptoms 24 h after admission to the ICU. These findings validate*,* a posteriori, the decision to carry out decompressive hemicraniectomy as a salvation therapy in life-threatening situations, in patients with intractable high ICP and brain herniation. However, the long-term outcome of patients undergoing decompressive craniectomy was not markedly different from that of other patients, despite the initially life-threatening situation due to high ICP and brain herniation. One patient died, but one young patient displayed a spectacular reversal of symptoms after ICP was brought under control by decompressive craniectomy, with the unexpected outcome of a total absence of long-term neurologic complications. No post-surgery complications were observed, not even during intensive care nursing.

Finally, like published case reports, the results for our series support the notion that salvation decompressive hemicraniectomy should be offered to patients with HSE and high ICP not responding to medical treatments. Rapidly growing HSE lesions (due to local bleeding or edema) may act as space-occupying lesions, generating a high ICP and leading to death from temporal swelling with brainstem compression. From this pathophysiological point of view, the brain lesions observed in severe HSE closely resemble the intracranial hypertension observed in malignant middle cerebral artery (MCA) infarction but differ from that observed in cases of traumatic brain injury. Given the demonstrated benefit of decompressive hemicraniectomy [28, 29] in malignant MCA infarction, this suggests that decompressive craniectomy should be considered in cases of severe HSE with intractable high ICP due to temporal herniation of the brain. However, in cases of malignant MCA infarction, decompressive hemicraniectomy is generally proposed before the occurrence of brain herniation in patients selected according to strict criteria based on clinical evaluation and assessments of the volume of ischemic lesions on brain scans. These conditions cannot be extrapolated to HSE. The clinical symptoms of HSE are often nonspecific, delaying medical consultation. In our study, the median time from symptom onset to the first brain scan was 5 days. By contrast, the corresponding interval for patients with acute stroke is only about 10 h [30]. Moreover, the potential for predicting brain herniation from the first brain scan is low (50 % sensitivity). Clinicians cannot therefore reliably predict the occurrence of brain herniation and therefore anticipate the potential toned for decompressive craniectomy.

This study has several limitations. There is a risk of coding misuse and errors in administrative databases. However, we focused on major codes, which are less likely to be forgotten or misinterpreted (*herpes encephalitis*, *death, admission to ICU, mechanical ventilation*). We validated the performance of our algorithm and identified no cases of false-negative or false-positive HSE diagnosis. However, as HSE is a rare condition, the probability of finding a false negative (i.e., diagnosis of HSE recorded in the patient’s medical records but not picked up in the PMSI search) is low. This may have resulted in an overestimation of the negative predictive value and, thus, an underestimation of the true hospital incidence. The validation algorithm was performed on medical files of patients admitted to the university hospital, and not in the other general or private hospitals of the study. However, we previously showed that coding process for infectious diseases was homogenous and showed no difference between the different French hospitals included in this study [21, 23, 31, 32]. Moreover, a study in France showed that for encephalitis with a precise diagnosis code, concordance between coding approach and prospective data collection was acceptable [33]. The retrospective design and the small number of patients are also limitations of the monocentric part of the study, but these are key aspects of PMSI use [21–23]. These concerns are intrinsic to the very low incidence of this disease. Furthermore, there is currently no evidence to support the use of decompressive hemicraniectomy, and the use of this procedure differs considerably between centers, as reported in the case reports (Additional file 4). We therefore preferred to carry out a single-center study to avoid the potential problems of a center effect bias. Like any therapeutic procedure, hemicraniectomy for HSE should be evaluated in accordance with the current standards for clinical trials (randomized controlled trials). However, this would be very difficult due to the very low incidence of high ICP in HSE and the ethical and technical problems raised by such a trial. Finally, our study adds new evidence to the debate concerning the relevance of decompressive hemicraniectomy as a salvage therapy for HSE with intractable high ICP and life-threatening brain herniation. The use of the Glasgow Outcome Scale to assess recovery 1 year after the initial admission for HSE may also be questionable. It is a validated tool to evaluate outcome after neurological insult [34], but has some limitations, and notably, mild neuropsychological impairment might be underestimated [35]. However, as follow-up has not been standardized, we preferred to use a simple tool, feasible even for patients whom follow-up was provided only out of the university hospital.

## Conclusions

This new estimate reveals a higher incidence of HSE since the routine use of PCR for diagnosis. Furthermore, morbidity remains high after severe HSE, and the mortality associated with HSE remains important during the acute phase. Despite the limitations of our study, this population-based observational study provides the first evidence supporting the concept that decompressive hemicraniectomy could be considered in the therapeutic arsenal of severe HSE management with life-threatening elevated ICP. Our findings highlight the need to increase our knowledge of possible therapeutic strategies for HSE.

## Key messages

- The hospital incidence of adult herpetic encephalitis was 10 times higher than previously reported.
- Morbidity and mortality remain high during the acute phase, so intensive care specialists should promptly take charge of these patients.
- In the most severe cases, patients may have high intracranial pressure with temporal herniation.
- Initial brain images are not predictive of the risk of brain herniation.
- In life-threatening situations with intractable high intracranial pressure and temporal herniation of the brain, decompressive hemicraniectomy appears to be a useful salvage therapy that could be considered.

## Additional files

Additional file 1:
**Detailed characteristics of the patients admitted to ICUs for HSE during the study period.** (PDF 81 kb) Additional file 2:
**Characteristics and management of the three patients who underwent decompressive craniectomy.** (PDF 345 kb) Additional file 3:
**Comparison between brain imaging techniques used for the first brain imaging after admission.** (PDF 76 kb) Additional file 4:
**Literature review for adult case reports and series of patients undergoing decompressive hemicraniectomy for severe herpes simplex encephalitis.** (PDF 57 kb)

## Abbreviations

CSF: cerebrospinal fluid
CT: computed tomography
GCS: Glasgow Coma Scale
GOS: Glasgow Outcome Scale
GP: general practitioner
HSE: herpes simplex encephalitis
HSV: herpes simplex virus
ICD: International Classification of Diseases
ICP: intracranial pressure
ICU: intensive care unit
MCA: mid cerebral artery
MRI: magnetic resonance imaging
PCR: polymerase chain reaction
PMSI: *Programme de Médicalisation des Systèmes d’Information*
SOFA: Sequential Organ Failure Assessment
