# Supplementary material for: Long-term outcome of severe herpes simplex encephalitis: a population-based observational study
Source: Crit Care. 2015 Sep 21;19(1):345. doi: 10.1186/s13054-015-1046-y (PMC4576407; doi:10.1186/s13054-015-1046-y)
Supplement: Additional file 1: — Detailed characteristics of the patients admitted to ICUs for HSE during the study period. (PDF 81 kb) [file 13054_2015_1046_MOESM1_ESM.pdf]

Additional figure 1 - Detailed characteristics of the patients admitted to intensive care units for Herpes simplex encephalitis during the study period.

Patients		Age	BMI (kg/m^2) or Weight	Chronic medical condition	ICU admission motif	Vital parameters at admission			Biology at admission		Lumbar punction findings			First brain imaging	Mechanical ventilation	Use of sedatives (days)	Fluid resucitation during first 24h (L)	Vasopressors		Renal Replacement therapy	ICU stay (days)	Glasgow Outcome Scale at one year	Follow-up
						GCS	Temperature (°C)	Blood Pressure (mmHg)	WBC (G/L)	CRP (mg/L)	Leucocytes (/mm^3)	Lymphocyte (%)	Protein level (g/L)	delay from symptoms onset (days)	Duration (days)			Yes/no	Duration (days)				
No brain herniation No Decompressive Craniectomy	*1*	22	46kg	None	Seizure + coma	14	38,6	145/80	10,3	21,2	100	90	1,24	5	1	1	none	No	x	no	8	5	UH then GP
	2	77	NA	atrial fibrillation, chronic congestive heart failure	Seizure + coma	5	38	111/70	13,8	NA	3	NA	0,57	2	40	9	1	Yes	36	no	41	1	Non applicable
	3	82	26	Hypertension	coma	14	39	147/115	6,2	177	15	20	0,94	9	31	4	4	Yes	4	Yes (27 days)	44	4	UH
	4	42	32	None	Coma	14	38.6	178/93	10.1	3.4	96	59	2.43	4	6	1	0.5	No	x	No	24	3	GP
	5	34	17	None	Coma	14	36.9	138/72	6.4	<1	470	90	2.59	8	x	x	2	No	x	No	7	5	UH
	6	68	32	Asthma, Type II diabetes, obesity	coma	6	40	83/44	7.0	4.6	800	30	2.92	5	15	4	2	Yes	7	Yes (4 days)	23	4	GP
	7	57	20	Alcohol abuse	Coma	10	38.4	130/75	7.9	2.7	2300	95	0.70	1	6	0	none	No	x	No	10	4	UH
	8	71	22	Rheumatoid arthritis (prednisone and methotrexate)	Seizure + Coma	3	38.8	143/88	9.9	1,3	8	NA	0,51	4	3	1	1	No	x	No	3	5	UH then GP
	9	22	29	None	meningitis syndrome	15	36,9	141/81	7.7	<1	684	98	3.36	5	x	x	None	No	x	No	2	5	UH
	10	48	26	None	Delirium	15	38	168/84	14.3	23.6	110	100%	1.11	6	x	x	None	No	x	No	12	3	UH
	11	28	NA	None	meningitis syndrome	15	37.9	150/81	5.2	<1	69	98	0,64	3	x	x	None	No	x	No	6	5	UH
Temporal hernation & Decompressive Craniectomy	*12*	59	67kg	None	Coma	14	38	146/83	7.3	3.3	21	89	0,86	3	10	10	2	Yes	10	No	10	1	Non applicable
	13	31	16	None	Seizure + Coma	7	36.8	122/69	10.2	199	30	100	0,92	5	17	16	None	Yes	6	No	23	5	UH
	14	58	77kg	None	Coma	3	36.9	1145/80	9.6	22	24	30	4	7	19	6	1	Yes	9	No	25	3	UH

BMI: body mass index; GCS: Glasgow coma scale; GP: general practicioner; ICU: intensive care unit; NA: non avalaible; UH: university hospital; WBC: white blood count
