# Supplementary material for: Long-term outcome of severe herpes simplex encephalitis: a population-based observational study
Source: Crit Care. 2015 Sep 21;19(1):345. doi: 10.1186/s13054-015-1046-y (PMC4576407; doi:10.1186/s13054-015-1046-y)
Supplement: Additional file 2: — Characteristics and management of the three patients who underwent decompressive craniectomy. (PDF 345 kb) [file 13054_2015_1046_MOESM2_ESM.pdf]

Additional file 2

Characteristics and management of the three patients who underwent decompressive craniectomy

	**Patient A**	**Patient B**	**Patient C**
**Age**	56	31	58
**Initial symptoms**	influenza-like syndrome, disorientation and agitation, fever	Headache, fever	Headache, left hemiparesis, fever
**Inital radiologic findings**	hypodensities of right temporal lobe, right insulae, right frontotemporal region, and frontal lobe	bilateral temporal hypodensities	bilateral temporal and frontal hypodensities with moderate mass effect
**Date of occurrence of cranial hypertension**	Day 4	Day 11	Day 5
**Intracranial pressure monitoring**	No	Yes	No
**Date of decompressive craniectomy**	Day 6	Day 12	Day 5
**One year outcome**	GOS 1	GOS 5	GOS 3 (frontal syndrome)
**CT Scan**<br>Pre surgery	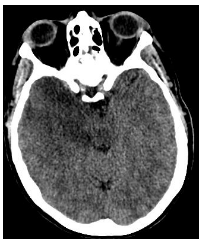	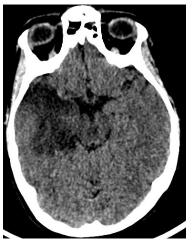	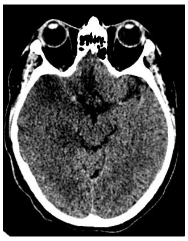
Post surgery	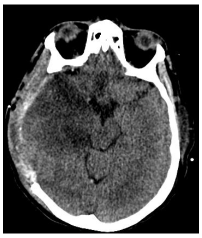	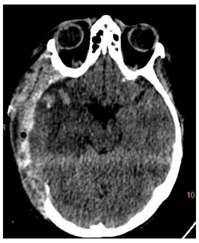	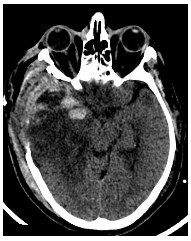
One year	Non available (death)	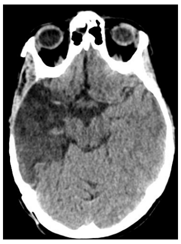	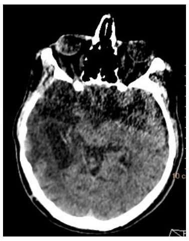

GOS: Glasgow Outcome Scale ; CT: computed tomography.
Dates are given in days after the onset of symptoms.
