# Supplementary material for: Long-term outcome of severe herpes simplex encephalitis: a population-based observational study
Source: Crit Care. 2015 Sep 21;19(1):345. doi: 10.1186/s13054-015-1046-y (PMC4576407; doi:10.1186/s13054-015-1046-y)
Supplement: Additional file 3: — Comparison between brain imaging techniques used for the first brain imaging after admission. (PDF 76 kb) [file 13054_2015_1046_MOESM3_ESM.pdf]

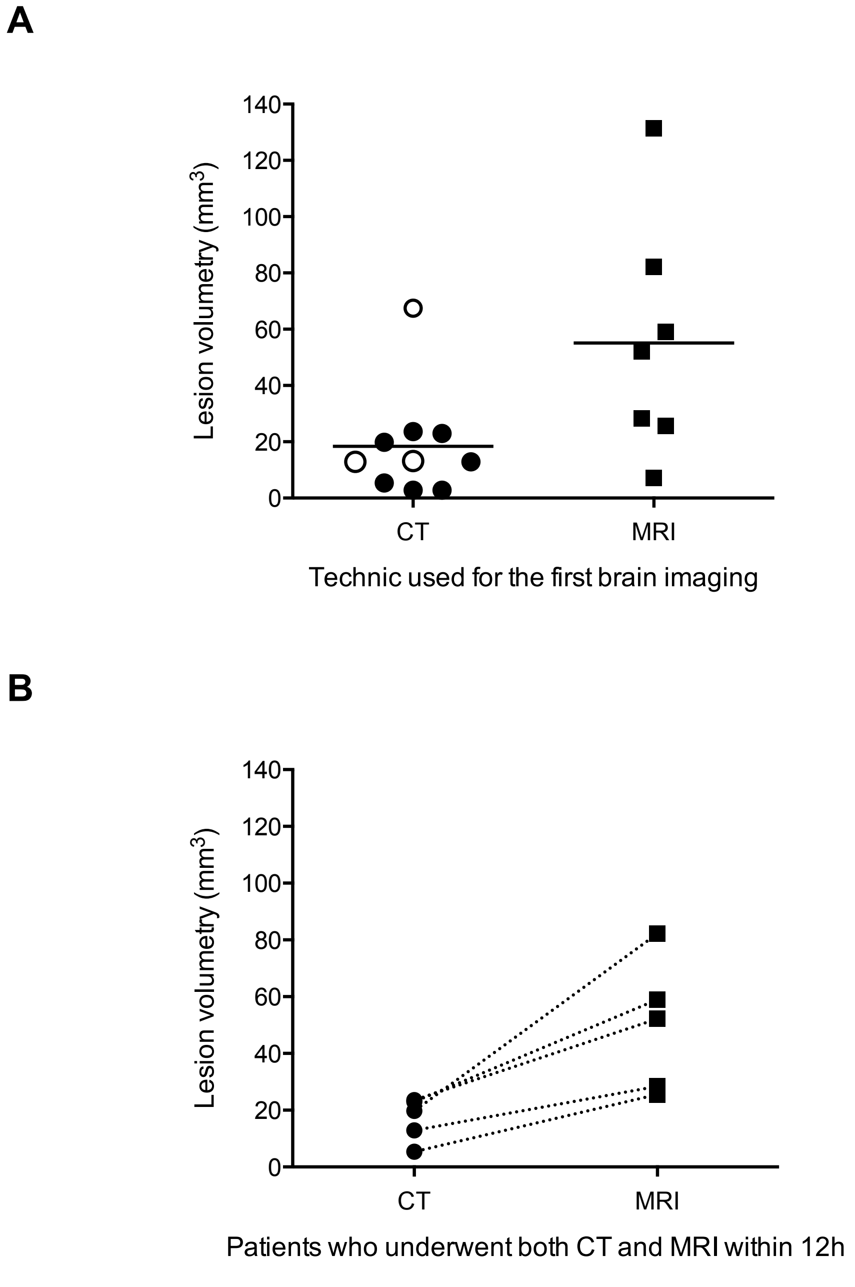

Additional file 3 - Comparison between brain imaging techniques used for the first brain imaging after admission. Brain lesion volumetry (mm^3) measured on the first brain image obtained, depending of the imaging technique used (computed tomography -CT- or magnetic resonance imaging -MRI-), for the entire cohort (**A**), and for paired procedures (**B**). Imaging procedures were considered to be paired if they were performed on the same patient within 12 h of each other. Patients with brain herniation and high intracranial pressure treated by decompressive craniectomy are represented as white dots.
