# Supplementary material for: Long-term outcome of severe herpes simplex encephalitis: a population-based observational study
Source: Crit Care. 2015 Sep 21;19(1):345. doi: 10.1186/s13054-015-1046-y (PMC4576407; doi:10.1186/s13054-015-1046-y)
Supplement: Additional file 4: — Literature review for adult case reports and series of patients undergoing decompressive hemicraniectomy for severe herpes simplex encephalitis. (PDF 57 kb) [file 13054_2015_1046_MOESM4_ESM.pdf]

Additional file 3 - Literature review for adult case reports and series of patients undergoing decompressive craniectomy for severe herpes simplex encephalitis

Author	Age (year), Gender (Male - M, Female -F)	Time from hospitalisation to DC (days)	Time from beginning of the symptoms to DC (days)	Herniation ?	Surgical technic	Complete Neurological recovery : Yes/No
**Schwab**	25, M	3	>3	Space occupying edema	Right hemispheric craniectomy	Yes
**Taferner**	42, M	NA	NA	Temporal herniation	Craniectomy + dural plasty + partial temporal lobectomy	Yes
	25, F	NA	NA	Temporal herniation	Craniectomy + dural plasty	Yes
**Yan**	48, F	5	13	Temporal herniation	Craniectomy + temporal lobectomy	Yes
	37, M	8/9	11	Temporal herniation	Craniectomy + temporal lobectomy	Yes
**Mellado**	21, F	1	9	Temporal herniation	Hemicraniectomy + dural plasty	No: frontal syndrome, aphasia, epilepsy
**Midi**	37, M	3	10	Temporal herniation	Craniectomy + anterior temporal lobe resection	yes
**Di Rienzo**	60, M	7	7	Temporal and transfalcine herniation	Craniectomy + dural plasty	Yes
**Adamo**	NA	<2	NA	Temporal herniation	Craniectomy + anterior temporal lobectomy + dural plasty	Yes
	NA	<2	NA	Temporal herniation	Craniectomy + anterior temporal lobectomy + dural plasty	Yes
**Gonzáles-Rabelino**	16, F	12	12	Temporal herniation	hemicraniectomy	Yes
**Maraite**	66, F	3	6	Temporal herniation	Hemicraniectomy + dural plasty	No: Memory troubles Barthel Index = 70
**Pili-Floury**	38, F	3	13	Temporal herniation	Hemicraniectomy + dural plasty	Yes

DC: decompressive craniectomy; NA: non available
